# Neonatal phthalate ester exposure induced placental MTs, FATP1 and HFABP mRNA expression in two districts of southeast China

**DOI:** 10.1038/srep21004

**Published:** 2016-02-12

**Authors:** Bin Li, Xijin Xu, Yueqin Zhu, Junjun Cao, Yuling Zhang, Xia Huo

**Affiliations:** 1 Laboratory of Environmental Medicine and Developmental Toxicology, and Provincial Key Laboratory of Infectious Diseases and Molecular Immunopathology, Shantou University Medical College, 22 Xinling Rd., Shantou, 515041, Guangdong, China; 2 The Second Affiliated Hospital of Shantou University Medical College, 69 Xiabei Rd., Shantou, 515041, Guangdong, China; 3 Department of Cell Biology and Genetics, Shantou University Medical College, 22 Xinling Rd., Shantou, 515041, Guangdong, China

## Abstract

Plastic production releases phthalate esters (PAEs), which can alter the expression of metallothioneins (MTs), fatty acid transport protein 1 (FATP1) and heart fatty acid binding protein (HFABP). A total of 187 mother-infant pairs were recruited, 127 from Chenghai (high exposed group) and 60 from Haojiang (low exposed group), to investigate the association between neonatal PAE exposure and mRNA expression of placental MTs, FATP1 and HFABP. Umbilical cord blood and placenta samples were collected for measuring five PAE concentrations and detecting mRNA levels of MTs, FATP1 and HFABP. Butylbenzyl phthalate (BBP), di(2-ethylhexyl) phthalate (DEHP), di-n-octyl phthalate (DNOP) were significantly higher in the high exposed group compared to the low exposed group. FATP1 and HFABP mRNA in the high exposed group were higher than that in the low exposed group while MT-1A was contrary. Both dimethyl phthalate (DMP) and DEHP were correlated with higher MT and MT-2A expression, while diethyl phthalate (DEP) was also positively correlated with MT-1A and FATP1 expression in female infants. DEHP exposure was negatively correlated with birth weight and gestational age in male infants. These results show that neonatal PAE exposure alters the mRNA expression of placental MTs and FATP1, which are related to fetal growth and development.

Chenghai is a district located in Shantou, Guangdong province, in southeast China. With more than 4,000 toy factories and 200,000 workers engaged in the plastic industry, Chenghai has established its reputation as a world-class toy manufacturing hub^1^. The large scale production of plastic goods may release huge amounts of chemicals such as phthalate esters (PAEs), into the environment, thereby impacting the health of workers and residents in Chenghai. The subjects of low exposed group were from Haojiang district, 29.2 km away from Chenghai. Haojiang is a tourism area with some parks and several shore resorts. The lifestyles and dietary habits of people living in this district are similar to that of Chenghai.

Phthalate esters (PAEs), including butylbenzyl phthalate (BBP), dimethyl phthalate (DMP), diethyl phthalate (DEP), di(2-ethylhexyl) phthalate (DEHP) and di-n-octyl phthalate (DNOP), are the most widely used synthetic plasticizers in the plastic production industry, as well as many other applications. PAEs are found in many commonly used products, including toys, cosmetics and inks. PAEs readily migrate from such products into environment, and they are resistant to natural degradation^2^. Humans are exposed to PAEs through ingestion, inhalation, dermal absorption and medical treatment during their lifetimes, including intrauterine life^3^,^4^. PAEs and their metabolites have been detected in various fluids of humans and animals, including peripheral and umbilical cord blood, breast milk, seminal fluid and urine^5^,^6^. Reproductive toxicity of PAEs has been confirmed in both humans and animals, and involves testicular toxicity, damage to spermatogonia and reduction of testosterone level^7^,^8^,^9^. PAEs also cause embryotoxicity and teratogenicity in animals, and have been reported to be endocrine disruptors^10^,^11^,^12^.

Metallothioneins (MTs) are known as important metal-binding proteins, for containing sulfur-based metal clusters that can bind Zn^2+^, Cd^2+^, and Cu^2+^ ions^13^. MTs are cysteine-rich, 6 to 7 kDa low molecular mass proteins, and are present in all organisms studied^13^,^14^. In mammals, four distinct MT isoforms, designated as MT-1 to MT-4. MT-1 and MT-2, are the most predominant isoforms, are widely expressed in almost all tissues, and are particularly abundant in liver and kidney. MT-3 is mainly found in brain and male reproductive organs, and MT-4 is especially located in stratified squamous epithelium^15^. With metal-binding properties, MTs coordinate many different metals, indicating MT isoforms may regulate micronutrient homeostasis and heavy metal detoxification^16^. Reports showed MTs bind and transfer free zinc directly to zinc-dependent enzyme, which suggests that MTs serve as a transportation medium and reservoir for zinc homeostasis^15^,^16^. Because of their important functions as metal regulators, altered expression of MTs may disturb micronutrient homeostasis or weaken heavy metal detoxification.

Fatty acid transport protein 1 (FATP1) and heart fatty acid binding protein (HFABP), expressed in placenta, are involved in the transfer of essential fatty acid between the mother and the fetus, which plays an important role in fetal development^17^. A previous study demonstrated significant changes in the expression of FATP1 and HFABP in the rat placenta upon maternal exposure to DEHP^18^.

Thus, alterations in placental MTs, FATP1 and HFABP mRNA may pose an adverse influence on the fetal health. Different from animal models, human exposure is more complicated and few studies *in vivo* have explored the corresponding mechanism. The aim of this study was to investigate possible associations between concentrations of five PAEs in umbilical cord blood and expression of MT, MT-1A, MT-2A, FATP1 and HFABP mRNA from homologous placenta.

## Results

### Demographic characteristics of the subjects

The demographic characteristics of subjects enrolled in this study were analyzed. Gestational age in the high exposed group was shorter than that in the low exposed group (Table 1; *P* = 0.005). Comparison of birth outcomes between two groups shows that birth weight (*P* = 0.019), length (*P* = 0.049) and Apgar score (*P* = 0.040) were lower in the high exposed group (Table 1).

### PAE exposure outcomes

This study measured five PAEs in umbilical cord blood samples, including BBP, DEP, DEHP, DMP and DNOP. These congeners are the most commonly used phthalate esters. DEHP levels were the highest of the five phthalates measured in both groups, followed by DEP. In the high exposed group, levels of BBP (*P* = 0.032), DEHP (*P* = 0.003) and DNOP (*P* < 0.001) were higher than those in the low exposed group (Table 1).

### Placental expression of MTs, FATP1 and HFABP mRNA

The mRNA expression levels were determined by quantitative reverse transcriptase PCR. As shown in Table 2, MT-1A mRNA (*P* = 0.047) was higher in the low exposed group compared with that in the high exposed group. Expression of FATP1 (*P* < 0.001) and HFABP (*P* < 0.001) mRNA in the placenta from the high exposed group were 63% and 178% higher than those in the low exposed group, respectively (Table 2).

### Association between PAE levels with birth outcomes, mRNA levels

After adjustment for group and gender, multiple regression analysis was used to calculate the association between PAEs with gestational age and birth weight (Table 3). As there is a strong relationship between gestational age and birth weight, we also adjusted gestational age in the coefficient analysis between PAEs and birth weight. DMP levels negatively correlated with gestational age (*β* = *−*0.556, *P* = 0.038), whereas DNOP positively correlated with gestational age (*β* = 0.685*, P* = 0.035). BBP positively correlated with birth weight (*β* = 0.587, *P* = 0.012), but not with gestational age. In the high exposed group (Table 4), correlation between DEHP and MT-1A mRNA was positive (*r*_*s*_ = 0.274, *P* = 0.005). DNOP negatively correlated with both MT (*r*_*s*_ = −0.265, *P* = 0.004) and MT-2 A (*r*_*s*_ = −0.256, *P* = 0.005). FATP1 and HFABP mRNA levels were increased as DEP levels increased (*r*_*s*_ = 0.312, *P* = 0.001; *r*_*s*_ = 0.243, *P* = 0.010). Considering gender could possibly also be an effect modifier for the observed associations, we divided all the samples into male and female. Covariates, including mothers’ age, mothers’ education, consumption of tea, fish, beans and milk, and fish oil supplement, were adjusted in the analysis. Exposure to DEHP resulted in decreased weight (*β* = −0.414, *P* = 0.021) and gestational age (*β* = −0.497, *P* = 0.004) in male infants (Table 5). The expression of MT-2A was inversely associated with length (*β* = −0.311, *P* = 0.042) in male infants, whereas the expression of MT-1A was inversely associated with both weight (*β* = −0.396, *P* = 0.041) and length (*β* = −0.415, *P* = 0.031) in female infants (Table 6). As shown in Table 7, DMP induced the expression of MT (*β* = 0.470, *P* = 0.012) and MT-2 A (*β* = 0.497, *P* = 0.007) in male infants, with similar findings obtained in female infants (*β* = 0.711, *P* = 0.003; *β* = 0.754, *P* = 0.001). There was a positive correlation between DEHP and MT (*β* = 0.571, *P* = 0.026), as well as DEHP and MT-2 A (*β* = 0.622, *P* = 0.026) in female infants. DEP was positively correlated with HFABP (*β* = 0.576, *P* = 0.001) in male infants, and MT-1 A (*β* = 0.561, *P* = 0.030) and FATP1 (*β* = 0.559, *P* = 0.030) in female infants.

## Discussion

In this study, we report the correlation between five PAEs and the mRNA expression of MT, MT-1A, MT-2A, FATP1 and HFABP. This study indicates potential risks of PAE exposure. Although MTs are thought to govern the regulation of metals, this research shows that neonatal PAE exposure can alter expression of MTs^19^. The association between neonatal PAE exposure with FATP1 mRNA expression was observed in this study, which supports an effect of PAE exposure on placental essential fatty acid homeostasis.

Due to widespread use of PAE-containing materials, the populations in non-industrialized zones could be easily exposed to several harmful industrial organic chemicals from many sources. It has been reported that people can be exposed to PAEs when plastics produced with PAE-containing plasticizers are used^20^,^21^. PAEs added in building materials can attach to and be transported by indoor dust leading to PAE exposure. Although all five PAEs were detected from both groups, three PAE congeners were significantly higher in the high exposed group. We speculate that the elevated PAE levels in the high exposed group may be directly caused by contaminating PAEs released from toy manufacturing, due to the large volumes production and lack of protection strategies^1^.

DEHP levels were the highest of the five phthalates measured in both groups, which corresponds to its wide used as an additive in the plastic matrix^20^,^21^. DEHP is one of the most frequently used phthalates, and it accounts for approximately 50% of total plasticizer production^22^. A study in Taiwan found that in 1200 food stuffs, DEHP was the predominant compound, and that it was the compound which had the highest average daily dose (ADD)^23^. Mihir *et al.* also found that DEHP was the most abundant, after detection and quantification of 15 phthalate esters in different exposure conditions in the South Delhi region^24^. For male infants, the decreased birth weight and gestational age were associated with increasing DEHP exposure. After controlling for mothers’ age and education level (but not controlled for dietary habits), the gestational age was still shorten with the DEHP (*β* = −0.332, *P* = 0.045, data not shown in tables). This is consistent with a prior study investigating environmental PAE exposure and preterm birth in Boston, the preterm births were found to be associated with increased intrauterine inflammation by PAE exposure^25^. Besides a direct adverse effect resulting from DEHP exposure, the decreased birth weight observed here could also be due to the shorter gestational age, although the dietary nutrient supplement remedied the weight decrease. Likewise, in another study, maternal supplementation was found to counteract the adverse effects of phthalate exposure in the human fetus^26^.

DMP induced the mRNA expression of MT and MT-2 A in both male and female infants. Moreover, expression of MT and MT-2A mRNA were also induced by DEHP in female infants, suggesting that different MT isoforms could be affected by different PAE congeners. However, over expression of MT isoforms appeared to be not good for fetal growth, since MT-2A expression was negatively associated with the birth length of male in this study. For female infants, the birth weight and length were also reduced as MT-1A expression increases. In the Spearman correlation analysis for genes and exposure factors, induction of MT-1A mRNA positively correlates with DEHP levels. On the other hand, DNOP was negatively correlated with expression of MT and MT-2A mRNA. Although DEHP and DNOP are congeners, the opposing correlations might be due to the different exposure concentrations, or chemical structure. Moreover, it was similar in the gender grouping analysis, which indicates that the gender of neonate is one of the important factors during the exposure period. While varied factors react to the extensive mix of PAEs, it is too complicated to specify a single-track causality. Animal studies revealed that DEHP exposure resulted in up-regulation of MT-1A and MT-2A in maternal liver, whereas both proteins were down-regulated in the embryo head^19^. Separate from their ability to directly bind metals, some metallothionein isoforms have antioxidant functions^15^, which may be the reason that MT mRNA expression is altered by exposure due to the oxidizability of PAEs. Early studies pointed out that MT could be activated by oxidative stress *in vitro* and *in vivo*^27^. In the case of organic chemicals, MT-Zn may release less Zn^2+^ to defend against oxidative stress from hydrogen peroxide^28^. Therefore, Zn^2+^ may be released to counter oxidative stress, leading to a breakdown in zinc homeostasis and insufficient zinc for some specific proteins.

Xu *et al.* showed that DEHP and its metabolites could change the expression of proteins involved in essential fatty acid homeostasis, and that the expression of PPAR, PPARγ, FAT/CD36, FATP1, and HFABP were up-regulated^17^,^29^. We find that expression of FATP1 and HFABP in high exposed group was elevated in people residing in environments contaminated by plasticizers. The association between DEHP exposure and essential fatty acid homeostasis in humans, reported in the present study, is consistent with the previously reported laboratory animal studies. DEHP and its metabolites influence essential fatty acid transfer across HRP-1 cells, implying that these compounds may alter placental essential fatty acid homeostasis and potentially result in abnormal fetal development^29^.

FATP1 and HFABP expression were less in the low exposed group, but there was no difference between male and female infants (data not shown). FATP1 expression was positively associated with birth weight in male infants, but it was not observed in female infants. For female infants, there were positive correlations between DEP and MT-1A, DEP and FATP1, while MT-1A was related to the reduced birth weight and length. Compared with male infants, induced FATP1 expression appears to have less contribution to birth weight. The altered essential fatty acid transfer may partly result from DEHP-mediated alterations in trophoblastic expression of lipid/fatty acid homeostasis-regulating proteins. A rise in the uptake and transport of selected fatty acids and accumulation of various fatty acid/lipid classes in the placenta, may lead to higher placental metabolism/lipid synthesis, and thus reduce the fetal supply of essential fatty acids, leading to adverse fetal development^17^. Over-expression of MTs may lead to disorders of micronutrient homeostasis, whereas inhibition of MT expression may cause failure to detoxify heavy metal ions in a multi-pollutant exposure environment, both of which may affect fetal growth. Exposure to PAEs during the pregnancy influences the expression of FATP1 and HFABP mRNA levels in the placenta, which may indirectly affect fetal growth and development. This effect differs from male to female infants.

In conclusion, the present study shows that PAE contamination is one of the main sources of environmental pollution in Chenghai and that neonatal PAE exposure can alter the mRNA expression of placental MTs and FATP1, which are related to fetal growth and development. These results contribute to further research of the adverse effects of PAE exposure on fetal growth and development.

## Methods

### Study population and sample collection

The Human Ethical Committee of Shantou University Medical College have approved our experiments. Informed consent forms have been obtained from all subjects and methods were carried out in accordance with the approved guidelines. A total of 187 mother-infant pairs were recruited, 127 from Chenghai (high exposed group) and 60 from Haojiang (low exposed group). All participants received a questionnaire to obtain information on demographic characteristics. Umbilical cord blood and placenta were collected by trained nurses. Neonatal physiological indices of birth weight, head circumference, body length and Apgar score were measured by medical professionals after delivery.

Umbilical cord blood (25 to 30 mL) was collected shortly after delivery by drawing with a stainless steel needle into one EDTA (ethylenediaminetetraacetic acid)-containing and four EDTA-free evacuated glass tubes. All samples were transferred on ice to the laboratory. After coagulation, EDTA-free samples were centrifuged (2,000 rpm, 15 min) at room temperature to obtain serum which was stored at −20 °C until the detection of PAEs. All the glass materials and containers were washed and soaked in 10% (v/v) HNO_3_ (guaranteed reagent, Donghong, Guangzhou, China) and received heat treatment in a muffle furnace at 400 °C for 4 h before use. Volumetric glassware that could not be heated was thoroughly rinsed with hexane (pesticide grade; TEDIA, USA).

After delivery, a small piece of placental tissue (1 cm × 1 cm), from both the maternal and fetal side of the placenta was cut and deposited in a freezing tube for storage in liquid nitrogen by midwives. All tubes were kept at −70 °C after being transported to the laboratory.

### Chemical analysis of PAEs in umbilical cord serum

The procedure described by Colón *et al.* was used with minor modifications^30^. Each serum sample (2 mL) was extracted with 10 mL of acetone/hexane (1:1, v/v; pesticide grade; TEDIA, USA) by vortexing for 30 min. The resulting solution was transferred to a separating funnel, shaken for 1 min, and then allowed to separate. The top organic layer was collected and the remaining sample was re-extracted using the same procedure. The two extracts were then combined and the solution was evaporated under N_2_ flow until nearly dry. Then 0.1 mL hexane was added, and the dissolved residue was transferred to a glass insert in amber GC vials.

Samples were analyzed with an Agilent 7890 A-5975 C gas chromatograph/mass spectrometer (Agilent Technologies, USA), with electron ionization used in the selected ion monitoring mode. Auto splitless injection onto a J&W Scientific DB-5 MS (30 m × 0.25 mm i.d., 0.25 μm film thickness) capillary column was used for the determination of phthalate congeners, with helium (1 mL/min) used as the carrier gas. The ion source, quadrupole and interface temperatures were set to 230 °C, 150 °C, and 280 °C, respectively. Retention times for phthalate congeners were established according to standards. Ion fragments at 91, 149 and 163 m/z were monitored for phthalate congeners. Oven temperature was ramped as follows: 80 °C for 2 min, increased to 230 °C at 15 °C/min and held for 5 min, then increased to 280 °C at 15 °C/min and finally held for 10 min. Because of the ubiquitous presence of phthalates, we analyzed adequate sets of analytical blanks before sample analysis, including procedural and solvent blanks, as well as blanks for the sampling device, pipettes and storage tubes, to reduce the laboratory contamination to the barest minimum. The detection limit was 0.02 μg/L to 0.06 μg/L. Recovery rates varied from 80.3% to 93.0%. Phthalate ester concentrations were calculated by comparison of peak areas with six point calibration curves (*r*^*2*^ > 0.99) constructed using an EPA phthalate ester mix (Sigma-Aldrich, Germany).

### Analysis of mRNA expression

Total RNA was isolated from 100 mg placental tissue using Trizol reagent (Invitrogen, USA) according to the manufacturer’s instructions. The quantity of RNA extracted was determined by OD_260_ measurement and RNA (1 μg) was reverse-transcribed to cDNA by using random primers. Quantitative PCRwas performed using a 7300 thermocycler (ABI, USA) and SYBR Green I (Takara, Japan) to detect synthesized products. The PCR was initiated at 95 °C for 30 s, followed by 40 cycles of denaturation at 95 °C for 5 s, while annealing and extension was at 60 °C for 30 s. Each sample was run with the internal control gene (β-actin and GAPDH) and relative expression levels of mRNA were calculated using the ΔΔCt method^31^. The primers were designed by Sangon Biotechnology Limited Company, Shanghai, China, and the sequences are as follows:

5′-ATGGATCCCAACAACTGCTCCTGC-3′ (upper) and

5′-AGGGCTGTCCCAACATCATCAGGC-3′ (lower) for MT;

5′-CT CGAAATGGACCCCAACT-3′ (upper) and

5′-ATATCTTCGAGCAGGGCTG TC-3′ (lower) for MT-1 A;

5′-CCGACTCTAGCCGCCTCTT-3′ (upper) and 5′-GTGGAAGTCGCGTTCTTTACA-3′ (lower) for MT-2A;

5′-TGACGTGGACATCCGCAAAG-3′ (upper) and

5′-CTGGAAGGTGGACA GCGAGG -3′ (lower) for β-actin;

5′-CTGCCCTTAAATGAGGCAGTCT-3′ (upper) and

5′-ACAGCTTCAGAGGGCGAAG -3′ (lower) for FATP1;

5′-GGGCACCTGGAAGCTAGTG-3′ (upper) and

5′-TTCGATGATTGTGGTAGGCTTG -3′ (lower) for HFABP;

5′-AGGTGAAGGTCGGAGTCA-3′ (upper) and

5′-GGTCATTGATGGCAACAA -3′ (lower) for GAPDH. We analyzed mRNA levels of placenta from 183 mother-infant pairs (high exposed group, n = 124; low exposed group, n = 59).

### Statistical analysis

Data were expressed as mean ± SD and median (25^th^–75^th^). The Mann-Whitney *U* test was used to analyze the non-normal distribution of phthalate data, mRNA expression and demographic characteristics. Multiple linear regression models were used to analyze the association between PAEs and both birth weight and gestational age. Spearman correlation analysis was used to explore potential associations between phthalate concentrations and related factors. Statistical analyses involved the use of SPSS v13.0 software (SPSS Inc., Chicago, IL, USA). The alpha level for all tests was 0.05, and the tests were two-tailed, *P* < 0.05 was considered statistically significant.

## Additional Information

**How to cite this article**: Li, B. *et al.* Neonatal phthalate ester exposure induced placental MTs, FATP1 and HFABP mRNA expression in two districts of southeast China. *Sci. Rep.* **6**, 21004; doi: 10.1038/srep21004 (2016).

## Figures and Tables

**Table 1:** Comparison of demographic characteristics and PAE concentrations between high and low exposed groups.

	High exposed group (n = 127)	Low exposed group (n = 60)	*P*
Mothers’ age (y)	26.19 ± 5.17	27.46 ± 4.70	0.073^a^
Gestational age (weeks)	38.72 ± 3.42 (33, 45)	39.38 ± 1.20 (37, 42)	0.005^a^
Baby birth weight (kg)	3.20 ± 0.43 (2.10, 4.60)	3.32 ± 0.41 (2.30, 4.30)	0.019^a^
Baby birth length (cm)	49.82 ± 3.45 (48, 56)	50.13 ± 1.64 (38, 52)	0.049^a^
Apgar score	9.91 ± 0.44	10.00 ± 0.00	0.040^a^
DMP (μg/L)	3.09 (1.79–5.00)	3.00 (1.16–4.40)	0.187^a^
DEP (μg/L)	258.67 (187.26–318.52)	247.51 (181.00–24.62)	0.381^a^
BBP (μg/L)	24.02 (19.42–33.68)	22.86 (18.31–24.62)	0.032^a^
DEHP (μg/L)	648.59 (433.80–854.15)	492.76 (403.17–635.66)	0.003^a^
DNOP (μg/L)	10.06 (7.79–12.72)	7.15 (5.33–11.08)	<0.001^a^

PAEs, phthalate esters; DMP, dimethyl phthalate; DEP, diethyl phthalate; BBP, butylbenzyl phthalate; DEHP, di(2-ethylhexyl) phthalate; DNOP, di-n-octyl phthalate.

Demographic characteristics are expressed as mean ± standard deviation, gestational age, birth weight and length are also expressed (minimum, maximum) and PAE concentrations are expressed as median (25^th^–75^th^).

^a^Analysis in Mann-Whitney *U* test.

**Table 2:** Comparison of MTs, FATP1 and HFABP mRNA expression between high and low exposed groups.

	High exposed group (n = 124)	Low exposed group (n = 59)	*P*
	Median (25^th^–75^th^)	Median (25^th^–75^th^)	
MT	0.1163 (0.0807–0.2186)	0.1432 (0.0803–0.2516)	0.488^a^
MT–1A	0.0057 (0.0033–0.0099)	0.0072 (0.0038–0.0142)	0.047^a^
MT–2A	0.0967 (0.0436–0.1942)	0.1028 (0.0572–0.1888)	0.814^a^
FATP1	0.0886 (0.0485–0.1672)	0.0543 (0.0271–0.0780)	<0.001^a^
HFABP	0.0874 (0.0310–0.1907)	0.0314 (0.0174–0.0589)	<0.001^a^

MT, metallothionein; FATP1, fatty acid transport protein 1; HFABP, heart fatty acid binding protein.

^a^Analysis in Mann-Whitney *U* test.

**Table 3:** Multiple linear regression analysis for the association between PAEs and both birth weight and gestational age.

	Birth weight^a^		Gestational age^b^	
	*β*	*P*	*β*	*P*
DMP	−0.211	0.465	−0.556	0.038
DEP	−0.178	0.406	0.504	0.142
BBP	0.587	0.012	0.565	0.061
DEHP	0.075	0.696	0.470	0.125
DNOP	0.324	0.132	0.685	0.035

DMP, dimethyl phthalate; DEP, diethyl phthalate; BBP, butylbenzyl phthalate; DEHP, di(2-ethylhexyl) phthalate; DNOP, di-n-octyl phthalate.

^a^Adjusted for group, gender and gestational age.

^b^Adjusted for group, gender.

**Table 4:** Spearman correlation analysis between PAEs and expression of MTs, FATP1 and HFABP mRNA in the high exposed group.

	MT		MT-1A		MT-2A		FATP1		HFABP	
	*r*_*s*_	*P*	*r*_*s*_	*P*	*r*_*s*_	*P*	*r*_*s*_	*P*	*r*_*s*_	*P*
DMP	0.061	0.501	0.043	0.641	0.164	0.071	0.032	0.673	−0.048	0.528
DEP	0.136	0.211	0.048	0.664	0.224	0.038	0.312	0.001	0.243	0.010
BBP	0.008	0.932	0.008	0.929	0.035	0.710	−0.004	0.962	−0.037	0.635
DEHP	0.002	0.842	0.274	0.005	0.041	0.683	0.058	0.464	0.048	0.558
DNOP	−0.265	0.004	−0.076	0.413	−0.256	0.005	−0.033	0.665	−0.046	0.665

DMP, dimethyl phthalate; DEP, diethyl phthalate; BBP, butylbenzyl phthalate; DEHP, di(2-ethylhexyl) phthalate; DNOP, di-n-octyl phthalate; MT, metallothionein; FATP1, fatty acid transport protein 1; HFABP, heart fatty acid binding protein.

**Table 5:** Multiple linear regression analysis for the associations between PAEs and birth outcomes of male and female infants.

		Male infants			Female infants		
		Birthweight^a^	Birthlength^a^	Gestationalage^a^	Birthweight^a^	Birthlength^a^	Gestationalage^a^
DMP	*β*	−0.300	−0.191	−0.311	0.244	−0.241	−0.011
	*P*	0.101	0.302	0.089	0.380	0.387	0.969
DEP	*β*	−0.291	0.009	−0.066	−0.113	−0.134	0.407
	*P*	0.112	0.964	0.723	0.688	0.634	0.132
BBP	*β*	−0.024	−0.081	0.056	0.604	0.190	−0.438
	*P*	0.899	0.665	0.766	0.017	0.497	0.103
DEHP	*β*	−0.414	−0.217	−0.497	0.308	0.069	0.162
	*P*	0.021	0.241	0.004	0.265	0.806	0.563
DNOP	*β*	−0.027	−0.068	0.053	0.603	0.280	−0.468
	*P*	0.885	0.714	0.778	0.017	0.312	0.078

^a^Adjusted for mother’s age, mother’s education situation, mother’s consumption of tea, fish, beans and milk, and fish oil supplements.

**Table 6:** Multiple linear regression analysis for the associations between mRNA expression and birth outcomes in male and female infants.

		Male infants			Female infants		
		Birthweight^a^	Birthlength^a^	Gestationalage^a^	Birthweight^a^	Birthlength^a^	Gestationalage^a^
MT	*β*	−0.175	−0.260	−0.261	0.011	−0.138	−0.111
	*P*	0.260	0.092	0.091	0.958	0.494	0.581
MT−1A	*β*	−0.136	0.102	−0.265	−0.396	−0.415	0.133
	*P*	0.385	0.515	0.086	0.041	0.031	0.510
MT-2A	*β*	−0.156	−0.311	−0.269	−0.068	−0.246	−0.043
	*P*	0.318	0.042	0.081	0.735	0.216	0.831
FATP1	*β*	0.319	0.015	0.258	0.006	0.113	0.114
	*P*	0.037	0.922	0.095	0.976	0.573	0.570
HFABP	*β*	−0.035	−0.008	0.061	−0.282	−0.179	0.126
	*P*	0.826	0.959	0.696	0.154	0.372	0.530

^a^Adjusted for mother’s age, mother’s education situation, mother’s consumption of tea, fish, beans and milk, and fish oil supplements.

**Table 7:** Multiple linear regression analysis for the associations between PAEs and expression of mRNA in male and female infants.

		Male infants					Female infants				
		MT^a^	MT-1A^a^	MT-2A^a^	FATP1^a^	HFABP^a^	MT^a^	MT-1A^a^	MT-2A^a^	FATP1^a^	HFABP^a^
DMP	*β*	0.470	−0.030	0.497	−0.342	0.073	0.711	0.420	0.754	0.065	−0.177
	*P*	0.012	0.880	0.007	0.075	0.711	0.003	0.119	0.001	0.819	0.528
DEP	*β*	−0.091	−0.373	−0.081	0.248	0.576	0.362	0.561	0.380	0.559	0.124
	*P*	0.646	0.051	0.681	0.203	0.001	0.185	0.030	0.163	0.030	0.660
BBP	*β*	−0.097	−0.096	−0.101	−0.174	−0.145	0.087	−0.367	0.037	−0.036	−0.092
	*P*	0.623	0.627	0.608	0.375	0.463	0.758	0.179	0.895	0.899	0.745
DEHP	*β*	0.092	0.202	0.102	−0.205	0.137	0.571	0.422	0.622	−0.169	−0.237
	*P*	0.641	0.301	0.605	0.296	0.488	0.026	0.117	0.013	0.547	0.395
DNOP	*β*	−0.104	−0.083	−0.109	−0.210	−0.154	−0.176	−0.285	−0.193	−0.238	-0.206
	*P*	0.597	0.675	0.582	0.283	0.435	0.531	0.304	0.490	0.394	0.461

^a^Adjusted for mother’s age, mother’s education situation, mother’s consumption of tea, fish, beans and milk, and fish oil supplements.
